# Understanding Experiential Satisfaction of M-Payment Apps During COVID-19 Pandemic

**DOI:** 10.3389/fpsyg.2022.893284

**Published:** 2022-04-25

**Authors:** Ratyuhono Linggarnusantra Putra, Margono Setiawan, Ananda Sabil Hussein, Agung Yuniarinto

**Affiliations:** Department of Management, Faculty of Economics and Business, University of Brawijaya, Malang, Indonesia

**Keywords:** perceived value, customer value, mobile payment, demography, satisfaction, COVID-19

## Introduction

Mobile payment is a method of payment through an application in mobile device. Mobile payment offers flexibility in which users can perform transactions anywhere and anytime (Dahlberg et al., 2008; Thakur and Srivastava, 2014; Tam and Oliveira, 2017), as users can deposit their money by transferring into their virtual account and use it for transactions. The transaction types of each user may vary due to various features of payment offered by mobile payment provider, such as buying goods, foods, or services, paying utility bills, and transferring money. Various steps and policies for handling the pandemic that led to social restrictions seemed to have also blocked business plans and movements. Like it or not, all business people must be ready to adapt to all changes. The existence of social restrictions has encouraged various business sectors to switch to digital transactions. In fact, there has also been a shift in people's behavior in carrying out transactions during the pandemic. The most concrete example is the rise of online transactions and the increasing adoption of mobile apps, namely, buying and selling practices that do not require face-to-face meetings. The context of social restrictions turns out to be very relevant to the digital transaction model.

To retain the existing users, the mobile payment providers are facing another challenge due to variance of users' demographic profile that can trigger certain preferences. By knowing their users' profile, it can be an advantage for the mobile payment providers to formulate appropriate strategies to ensure continuance intention to use among their users. The strategies hopefully help prevent their users to switch to another brand of mobile payment that fulfills the users' preferences or needs. For example, in terms of finances, mobile payment providers often offer benefits such as discount, cashback point, and subscription to attract both their existing and new users. The benefits can also be a critical factor to establish continuance intention to use for existing users who have concerns on financial related. As a technology product, ease of use can be considered as another determining factor for continuance usage, especially for people who are not tech-savvy. Thus, simplicity can be important to make them more effective in using the mobile payment application. According to the users' profile differences, each aspect offered by the mobile payment providers can affect the users at different levels. Thus, deep understanding on the existing users plays a key role to establish continuance intention to use among their users. This study was conducted in the Jakarta-Bogor-Depok-Tangerang-Bekasi (JaBoDeTaBek) area as the main area with high people mobility in Indonesia, where mobile payment users can find and experience many brands of mobile payment in the market, creating the challenge for mobile payment providers to retain their existing users.

In the marketing field, customer loyalty is considered as an important construct. Zhao and Kurnia (2011) mentioned that continuance intention to use is another meaning of loyalty that has been a significant element for user retention. Without spending money to attract new customers, company's profit can be increased through customer loyalty (Oliver, 2010). The statement is relevant with the phenomenon of competition among mobile payment providers nowadays, where they need to establish continuance intention to use among their existing users to ensure business sustainability. Continuance intention to use as a construct has ever been examined in some previous studies with different antecedents, such as perceived value (Chiu et al., 2014), satisfaction (Kim et al., 2009; Kuo et al., 2009; Zhou, 2012), and various perceived value dimensions, such as functional value, emotional value, social value, and monetary value (Wang et al., 2004; Deng et al., 2010; Kim et al., 2010).

Most of the previous studies found a significant impact of perceived value on continuance intention. However, some gaps still exist. The first gap shows that there is still inconsistency at the significance level between perceived value and continuance intention. Fiandari et al. (2018) found that the effect of perceived value on intention was not significant even though majority of studies showed significant effects between those constructs. To strengthen the significance level and make the relationship more consistent, experiential satisfaction was put as a mediating variable in this study. This study considered an opportunity for the inconsistency relationship to be addressed to be significant and more consistent through an appropriate mediating variable between perceived value and continuance intention to use. This study put experiential satisfaction as a mediating variable based on some previous studies (Cronin et al., 2000; Yang and Peterson, 2004; Lai et al., 2009).

The second gap is about the effect of demography on the significance level between perceived value and continuance intention. Different significance levels between two groups of respondents were found by Ryu (2017) in his study about Fintech. The study generated different significance levels between all respondents and categorized respondents, i.e., early adopters and late adopters. The significant result in all respondents changed to be insignificant in the early adopters category. The study concluded that the different significance levels were caused by different expectations and interests between consumers. However, demography, as a moderating variable between perceived value and continuance intention to use, has never been examined in previous studies, especially in the context of mobile payment continuance usage. Examining demography as a moderating variable will strengthen the predictive power of the research model through more comprehensive results that capture significance level differences between demography categories.

According to the research gaps, two research objectives were addressed in this study. The first objective is to examine direct effect of perceived value toward continuance intention to use, and indirect effect through experiential satisfaction as a mediating variable. The second objective is to examine demography categories, such as gender, age, economic class, and marital status, as moderating variables between perceived value, experiential satisfaction, and continuance intention to use. After the completion, this study provides both theoretical and practical contributions. For theoretical contribution, a new research model is developed by adding experiential satisfaction as a mediating variable and some demography categories as moderating variables to provide comprehensive results in capturing user's continuance intention to use mobile payment. For practical contribution, the results of this study can be a reference for mobile payment providers to understand the users' preferences as a basis to formulate strategies to establish continuance intention to use among their users.

## Research Model and Hypothesis Development

This section explains the proposed research model and hypotheses. The research model was developed based on consumption value theory to examine the relationship between perceived value and continuance intention to use. In addition, the research gaps in previous studies were considered to add another two constructs to enhance the predictive power of the model. The first construct is experiential satisfaction as a mediating variable between perceived value and continuance intention to use to strengthen relationship between those constructs, and the second construct is demography as a moderating variable between perceived value, experiential satisfaction, and continuance intention to use to capture the potential of significance level differences among demography categories of respondents.

## Discussion

**Figure 1:**
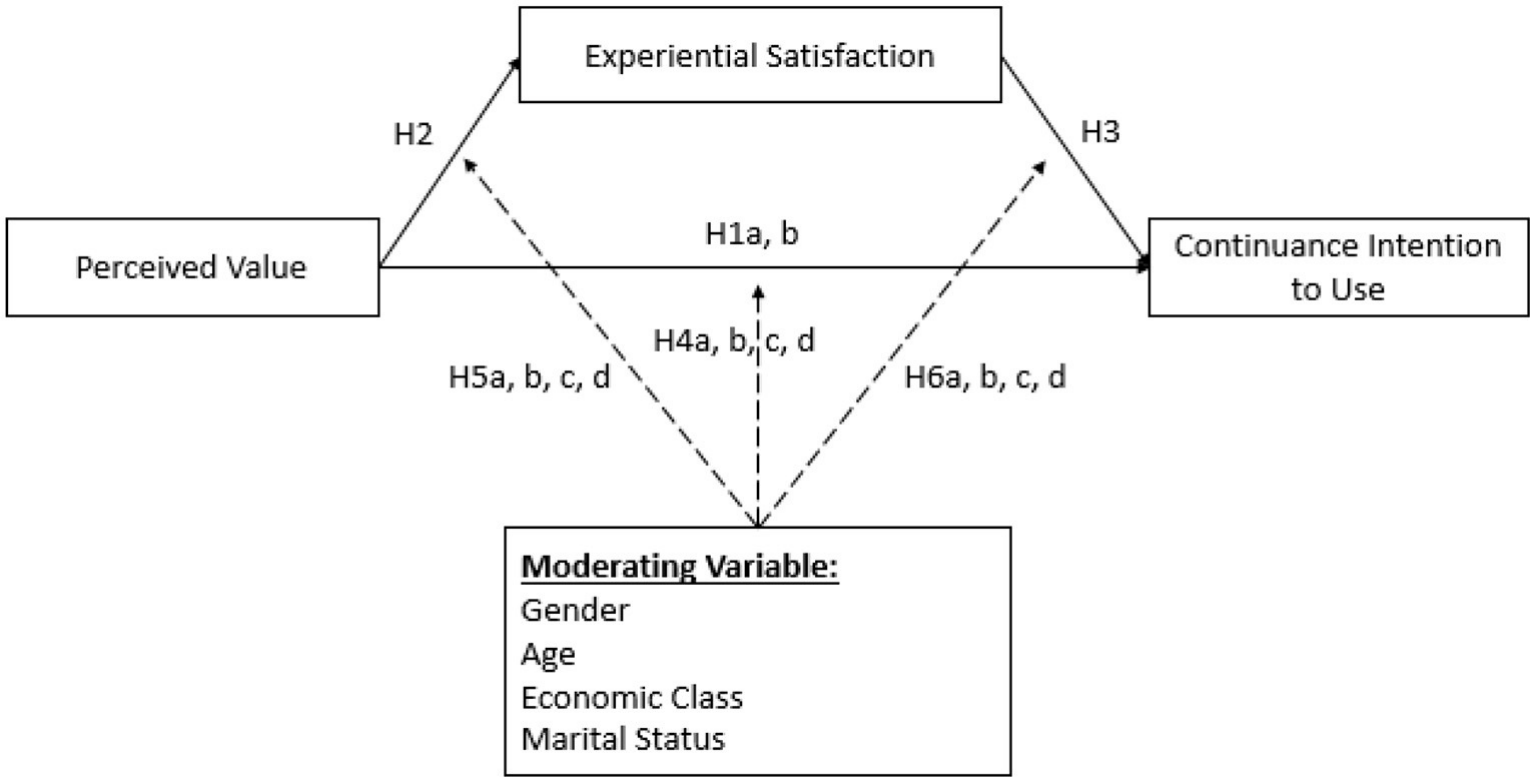
Conceptual model. 4a, The effect of gender as moderating variable on the effect of perceived value to continuance intention to use. 4b, The effect of age as moderating variable on the effect of perceived value to continuance intention to use. 4c, The effect of economic class as moderating variable on the effect of perceived value to continuance intention to use. 4d, The effect of marital status as moderating variable on the effect of perceived value to continuance intention to use. 5a, The effect of gender as moderating variable on the effect of perceived value to experiential satisfaction. 5b, The effect of age as moderating variable on the effect of perceived value to experiential satisfaction. 5c, The effect of economic class as moderating variable on the effect of perceived value to experiential satisfaction. 5d, The effect of marital status as moderating variable on the effect of perceived value to experiential satisfaction. 6a, The effect of gender as moderating variable on the effect of experiential satisfaction to continuance intention to use. 6b, The effect of age as moderating variable on the effect of experiential satisfaction to continuance intention to use. 6c, The effect of economic class as moderating variable on the effect of experiential satisfaction to continuance intention to use. 6d, The effect of marital status as moderating variable on the effect of experiential satisfaction to continuance intention to use.

This study has two research objectives. The first objective aims to examine the direct effect of perceived value to continuance intention and indirect effect through experiential satisfaction as a mediating variable, the model can be seen in Figure 1 for more details. The result showed that there is a significant effect of perceived value on continuance intention to use with 11.115 as t-statistic value. Thus, hypothesis 1a is accepted. This finding proved that perceived value is a crucial marketing construct that affects continuance intention to use. It supports findings in previous studies that found a significant effect of perceived value on continuance intention (Cronin et al., 2000; Bei and Chiao, 2001; Choi et al., 2004; Gallarza and Saura, 2006; Chen, 2008; Chen and Tsai, 2008; Ryu et al., 2008, 2012; Chen and Chen, 2010; Chen and Hu, 2010; Eid, 2015; Fahlevi and Alharbi, 2021). Furthermore, the result also showed that experiential satisfaction has a significant role as a mediating variable between perceived value and continuance intention to use. This means that hypothesis 1b is accepted, as can be seen from the score of Sobel-test, which is 10.219. This finding explains that perceived value has a stronger or more significant level to continuance intention to use indirectly through experiential satisfaction. It also means that experiential satisfaction can make stronger or more consistent the effect of perceived value on continuance intention to use. Thus, if mobile payment providers can ensure experiential satisfaction among their users while using the mobile payment, they will have a higher chance to retain users who will continuously use their mobile payment.

Moreover, regarding the relationship between perceived value and experiential satisfaction, the t-statistic value of 19.048 indicated that perceived value has a significant effect to experiential satisfaction. Thus, hypothesis 2 is accepted. It supports findings in previous studies that found a significant effect of perceived value on satisfaction (Choi et al., 2004; Gallarza and Saura, 2006; Lee et al., 2007; Chen, 2008; Chen and Tsai, 2008; Williams and Soutar, 2009; Wu and Liang, 2009; Chen and Chen, 2010; Deng et al., 2010; Eid, 2015; El-Adly and Eid, 2016; Fahlevi, 2021).

For the relationship between experiential satisfaction and continuance intention to use, the t-statistic value of 12.109 indicated that experiential satisfaction had a significant effect on continuance intention to use. Thus, the hypothesis 3 is accepted. It supports findings in previous studies that found a significant effect of experiential satisfaction on continuance intention to use (Tarn, 1999; Cronin et al., 2000; Bei and Chiao, 2001; Choi et al., 2004; Chen and Quester, 2006; Chen, 2008; Ryu et al., 2008, 2012; Deng et al., 2010; El-Adly and Eid, 2016).

The second research objective is to examine some demography categories as moderating variables between perceived value, experiential satisfaction, and continuance intention to use. The multi-group analysis results showed that gender, age, economic class, and marital status are significant moderating variables on the relationship between perceived value, experiential satisfaction, and continuance intention to use. Thus, all hypotheses related to moderating roles from demography categories are accepted.

The findings of this study provide both theoretical and practical contributions. For theoretical contributions, this study found the experiential satisfaction as a significant mediating variable between perceived value and continuance intention to use. Moreover, this study also found a significant moderating role from some demography categories, such as gender, age, economic class, and marital status, in the relationship between perceived value, experiential satisfaction, and continuance intention to use. Thus, this study is successful in more comprehensively establishing a comprehensive research model to capture perceived value effect to continuance intention to use as part of behavioral intention by adding experiential satisfaction as a mediating variable and demography as a moderating variable.

As for the research objectives, this study provides two practical implications. First, the significant result of perceived value and experiential satisfaction in enhancing continuance intention to use and the significant role of experiential satisfaction as mediating variable between perceived value and continuance intention to use explains that mobile payment provider needs to provide good value and experiential satisfaction for users to ensure continuance usage of their mobile payment. Second, findings on demography categories as moderating variable is important for the mobile payment providers to know their users' preferences more specifically in each demography category. This information can be used to formulate the company strategies to ensure continuance intention to use among their users.

## Conclusion

The findings in this study provide new insight for marketing academics and practitioners. Experiential satisfaction and demography are proven to be important constructs to increase the predictive power of previous research models that examined the relationship between perceived value and continuance intention to use, especially among mobile payment users. These findings explain that mobile payment providers should ensure to provide value and experiential satisfaction for their users that will enhance continuance intention to use. The higher level of continuous intention to use can be generated by providing high value and experiential satisfaction to their users. Nevertheless, there are several limitations that provide opportunities for future study. First, regarding the sampling design that only focused on mobile payment users in JaBoDeTaBek, Indonesia. This limitation implies that the results cannot be generalized to other settings with different cultures and characteristics. Second, the antecedent of continuance intention to use is limited to the perceived value with its dimensions and experiential satisfaction. Future research might include other important marketing constructs, such as perceived trust and perceived risk, for better prediction of continuance intention to use. Third, other demography categories can be tested as moderating variable to enrich information for mobile payment providers to formulate their strategies to ensure continuance intention to use among their users.

## Author Contributions

RP contributed to conceptualization, methodology, investigation, curation, analysis, funding acquisition, and writing. AH helped in investigation, curation, analysis, and writing. MS and AY helped in review, analysis, and writing. All authors contributed to the article and approved the submitted version.

## Conflict of Interest

The authors declare that the research was conducted in the absence of any commercial or financial relationships that could be construed as a potential conflict of interest.

## Publisher's Note

All claims expressed in this article are solely those of the authors and do not necessarily represent those of their affiliated organizations, or those of the publisher, the editors and the reviewers. Any product that may be evaluated in this article, or claim that may be made by its manufacturer, is not guaranteed or endorsed by the publisher.
